# Living beyond treatment: late effects and everyday life in Faroese breast cancer survivors

**DOI:** 10.3389/fonc.2026.1944286

**Published:** 2026-10-22

**Authors:** Beinta F. Glyn-Davies, Mariann Hansen, Paula K. Ólavsdóttir, Annika H. Davidsen

**Affiliations:** Faculty of Health Sciences, University of the Faroe Islands, Tórshavn, Faroe Islands

**Keywords:** breast cancer, cancer treatment, everyday life, late effects, small-scale societies, survivorship

## Abstract

**Background:**

Advances in breast cancer treatment have increased the number of survivors, many of whom experience persistent late effects beyond treatment completion. These late effects may significantly impact everyday life; however, limited research has explored survivors’ experiences of these challenges in small-scale societies such as the Faroe Islands.

**Aim:**

This study aimed to explore the late effects experienced by female Faroese breast cancer survivors following treatment, and how these impact their everyday lives.

**Methods:**

A qualitative study using Interpretative Phenomenological Analysis (IPA) was conducted. The authors’ reflexive approach to pre-understanding, openness, and dialogue was informed by Gadamerian hermeneutics. Seven breast cancer survivors in the Faroe Islands were recruited through cancer support networks and snowball sampling. Data were collected through two semi-structured focus-group interviews, one with five participants and one with two participants. The data were analyzed using IPA, with attention to individual meaning-making within the context of group interaction.

**Results:**

Four themes were identified: (1) Fatigue Sets the Agenda for Everyday Life, (2) Transitioning from Supported Patient to Abandoned Survivor, (3) A Disruption and Reconstruction of the Sense of Self, and (4) Invisible Late Effects and Social Misunderstanding. Survivorship was described as an ongoing and demanding process, characterized by persistent physical, emotional, and social challenges. Participants described late effects as embedded in everyday life, requiring continuous adaptation, renegotiation of identity, and management of social expectations. Limited follow-up support and insufficient recognition of invisible symptoms further complicated this process.

**Conclusion:**

Late effects following breast cancer treatment are multiple, complex, and may persist for weeks, months, and years after treatment completion. These findings highlight the need for increased information, recognition, and understanding of late effects and survivorship. Structured survivorship pathways, including individualized information, access to rehabilitation, continuity of follow-up across Faroese and Danish healthcare services, and a more gradual transition from the healthcare system, may better prepare survivors to navigate life after treatment independently.

## 1 Introduction

Breast cancer is the most frequently diagnosed cancer among women worldwide, including in the Nordic countries (1). In 2022, approximately 2.3 million women worldwide were diagnosed with the disease (2), and the prevalence of breast cancer continues to increase globally (3, 4). Breast cancer is among the most common types of cancer in the Faroe Islands (1). According to the NORDCAN database, approximately 29 patients were diagnosed with breast cancer per year in the Faroe Islands during the period 2019–2023 (5).

Breast cancer is a disease in which normal breast cells mutate, begin dividing uncontrollably, and form a malignant (cancerous) tumor. It most commonly develops in the epithelial cells lining the milk ducts. When cancer cells spread beyond the primary tumor to surrounding breast tissue, lymph nodes, or distant organs, the disease is classified as invasive and may metastasize through the lymphatic system or bloodstream. The most common symptom is a palpable lump in the breast or a swollen lymph node in the axillary region. In some cases, breast cancer remains asymptomatic in its early stages and is detected through routine screening procedures such as mammography (6).

Treatment for breast cancer most often begins with surgery. The surgical procedure may involve either removal of the tumor followed by reconstruction of the breast or removal of the entire breast (mastectomy). In cases of rapidly growing tumors, patients may receive neoadjuvant chemotherapy prior to surgery to reduce the extent of the surgical procedure. Treatment may also include chemotherapy, radiotherapy, endocrine therapy, and/or other systemic therapies, depending on the clinical indication following surgery. If the cancer has spread to the lymph nodes in the axilla, the affected lymph nodes are removed, and patients may receive physiotherapy to reduce discomfort in the axillary region (6).

Fifty years ago, Faroese patients were referred abroad to the Danish health care system for cancer treatment. However, breast cancer patients have in recent years received combined treatment shared between the Faroese and the Danish health care systems (7, 8). Since 2019, a Faroese specialist in oncology has been permanently appointed at the National Hospital of the Faroe Islands. In addition, specialist physicians regularly travel to the Faroe Islands to provide specialist care. This collaboration places greater demands on the quality of treatment and communication between the two countries to ensure sustainable and coherent treatment (7, 8).

Advances in breast cancer treatment and early diagnostics have significantly improved survival rates and have led to a growing population of breast cancer survivors. While improved survival represents a major public health achievement, it has simultaneously resulted in an increasing prevalence of late effects following treatment (3, 4). The American National Cancer Institute defines late effects as *“a health problem that occurs months or years after a disease is diagnosed or after treatment has ended. Late effects may be caused by cancer or cancer treatment. They may include physical, mental, and social problems and second cancers”* (9).

Late effects constitute a significant public health challenge, as they arise because of breast cancer or cancer treatment and may affect individuals across physical, psychological, and social domains. Such late effects may include radiation-induced injuries (e.g., burns), as well as sequelae related to surgery, chemotherapy, and endocrine therapy. They range from physical symptoms, such as fatigue and lymphedema, to psychosocial challenges, including anxiety, changes in identity, and difficulties in everyday life (3, 4). Women also report fatigue, pain, cognitive impairment, sleep disturbances, and neuropathy as the most challenging late effects of breast cancer treatment (10). Recent results from a Danish cohort study show that many patients diagnosed with breast cancer receive extensive, long-term treatment with curative intent. Hence, they become long-term survivors with a high risk of late effects (10).

Late effects are increasingly recognized as a distinct health concern, but many women still feel alone in dealing with the aftermath (11) – especially in geographically and medically peripheral regions (8). In the Faroe Islands – with its unique geography, culture, and healthcare structure (12) – access to follow-up support and specialized rehabilitation may be limited, which can affect how women experience living with late effects. However, there is limited knowledge about the presence of late effects among Faroese breast cancer survivors and how these are experienced in daily life.

This study therefore aims to explore the presence of late effects amongst female Faroese breast cancer survivors following cancer treatment, and their impact on everyday life. This study will use the definition of everyday life as *‘What happens every day, the routine, repetitive taken-for-granted experiences, beliefs and practices; the mundane ordinary world, untouched by great events and the extraordinary’* (13). This study seeks to generate knowledge that can inform and support the development of more targeted healthcare initiatives for breast cancer survivors in small-scale societies such as the Faroe Islands.

## 2 Materials and methods

### 2.1 Study design

A qualitative study design was used to explore Faroese breast cancer survivors’ experiences of late effects and their impact on everyday life. Data were collected through two semi-structured focus group interviews. This study was reported in accordance with the Consolidated Criteria for Reporting Qualitative Research (COREQ) 32-item checklist for interviews and focus groups. Interpretative Phenomenological Analysis (IPA) provided the methodological framework for the study and analysis (14). The authors’ reflexive approach to pre-understanding, openness, and dialogue was additionally informed by Gadamerian hermeneutics (15). Gadamer was therefore used as a sensitizing philosophical perspective on interpretation rather than as a separate analytic framework.

### 2.2 Participants and recruitment

A total of seven women participated in the study (see **Table 1** for participant characteristics). The inclusion criteria were women who had survived breast cancer, were 18 years or older and living in the Faroe Islands. For this study, completion of cancer treatment was defined as discharge from the oncology department following primary treatment, regardless of ongoing adjuvant endocrine therapy. Women undergoing active treatment, and women unable to participate due to health reasons were excluded.

**Table 1:** Overview of participant characteristics.

Participant characteristic	Description
Age at time of interview	49 - 68
Marital status	Married, n=6 Single, n=1
Children	Yes, n=6 No, n=1
Occupation	Part-time employment/independent entrepreneur, n=5 Early retirement, n=2
Year of breast cancer diagnosis	2010 – 2021. One participant did not report the year of breast cancer diagnosis.
Age at breast cancer diagnosis	40 – 58
Year of completion of primary treatment	2011 - 2024
Age at completion of primary treatment	41 – 59
Treatment modalities reported by participants	Breast surgery, n=7 Axillary lymph node surgery, n=7 Chemotherapy, n=6 Radiotherapy, n=6 Zoledronic acid (Zometa), n=2 Endocrine therapy, n=7

Participant characteristics are presented in aggregate to minimize the risk of deductive disclosure. Given the small population of the Faroe Islands, combining individual demographic, occupational, and treatment characteristics could increase the possibility of participant identification. This approach also reflects participants’ expressed wishes regarding confidentiality, as some participants specifically raised concerns about being recognized.

Recruitment was initially facilitated through the Faroese Cancer Society and a breast cancer support group; however, only three participants came forward through these channels. Recruitment was subsequently expanded using snowball sampling, which can facilitate access to populations that may be difficult to reach through conventional recruitment methods (16). One author encountered an eligible potential participant by chance in a public setting. The author knew the woman through her professional role as a nurse but had no personal relationship with her. Following information about the study, the woman offered to share the authors’ contact information with other breast cancer survivors in her network who might be interested in participating. Five additional women volunteered, through this process.

In total, eight women volunteered to participate. One was subsequently unable to attend due to work commitments, resulting in a final sample of seven participants.

### 2.3 Data collection

Data were collected through two semi-structured focus group interviews, each lasting approximately 120 minutes. One group consisted of five participants and the other two participants. The interviews were conducted in November 2025. Focus groups were chosen because they allow a group of individuals to interact and discuss a specific topic, aiming to draw from the personal experiences, beliefs, perceptions and attitudes of the participants through a moderated interaction (17). In this study, the format provided an opportunity for breast cancer survivors to reflect on, compare, and elaborate on their experiences of late effects and their impact on everyday life.

Focus groups commonly comprise six to eight participants; however, smaller groups of two to five participants may be appropriate when the participant pool is limited or difficult to reach and the research topic benefits from group discussion (17). Recruitment was challenging because of the limited pool of eligible participants within the small Faroese population and the low response to the initial recruitment strategy. Participants were also geographically dispersed, and the two interviews were therefore conducted close to participants’ places of residence to minimize travel. The second focus group was intended to include three participants; however, one participant was unable to attend due to work commitments. As the remaining two participants were present and willing to participate, the interview proceeded in a dyadic format.

The interviews were conducted in neutral and comfortable locations to support open discussion and minimize distractions (18). A moderator led each interview and encouraged all participants to contribute. Across the two interviews, the first three authors rotated between the roles of moderator, co-moderator, and observer.

### 2.4 Interview guide

An interview guide was prepared (see **Table 2**) to ensure consistency across the interviews. All questions were open-ended, allowing participants to share their experiences freely without being guided towards predetermined responses. The questions served primarily to initiate discussion, enabling participants to engage with one another, which is consistent with the principles of focus group interviews (19).

**Table 2:** Overview of interview guide.

Theme	Main questions	Sub-questions and supporting questions
Physical late effects	Have you experienced physical changes after the treatment ended?	Have the physical changes affected your daily life? If yes, how?
Psychological late effects	Have you experienced psychological changes after the treatment ended?	Have the psychological changes affected your daily life? If yes, how?
Social late effects	How have your relationships with family, friends, and work been after the illness?	What kind of support have you received? Have you missed support for anything specific?
Reflection and meaning	How have you found your own way of living with the late effects?	Have you experienced that your view of life or yourself has changed? Was there anything you missed after the treatment ended – or something that was especially helpful?
Conclusion	Is there anything that we have not talked about that you think is important to include.	What would you want healthcare professionals to know about life after breast cancer?

The interview guide was used in both focus groups and was not revised between interviews.

### 2.5 Data analysis

The data were analyzed using Interpretative Phenomenological Analysis (see **Table 3**), which draws on phenomenology, hermeneutics, and idiography to explore how people make sense of significant lived experiences (14). In IPA, interpretation involves a double hermeneutic, whereby participants seek to make sense of their experiences and the author, in turn, seeks to make sense of participants’ meaning making. Consistent with this interpretative position, the authors approached the analysis reflexively, recognizing that interpretation is shaped by prior understanding while remaining open to meanings expressed in participants’ accounts. Gadamerian concepts of pre-understanding and openness informed this reflexive stance but were not used as an additional or competing analytic framework.

**Table 3:** IPA data analysis process.

IPA step	Description	Application in this study
Step 1: Reading and re-reading	Thorough familiarization with the data through repeated engagement with participants’ accounts (20).	Transcripts were read several times to become familiar with each participant’s account, while initial thoughts and reflections were noted.
Step 2: Initial noting	Detailed exploratory comments addressing descriptive, linguistic, and conceptual aspects of the account (14).	All three authors produced detailed notes highlighting meanings, patterns, reflections, and interpretative observations for each participant.
Step 3: Developing themes	Identification and articulation of themes within each participant’s account while maintaining an idiographic focus (14).	Themes were developed for each participant based on the authors’ interpretations of the account before comparison across cases.
Step 4: Connecting themes	Examination of relationships and patterns between themes to develop a coherent account of the participant’s experience (14).	Connections between themes were explored iteratively, and related themes were grouped into broader patterns and superordinate themes.
Step 5: Moving to the next case	Repetition of the analytical process for each case while maintaining an idiographic focus (14).	Each participant’s account was considered individually before cross-case comparison, allowing attention to both individual experiences and shared meanings.
Step 6: Looking for patterns across cases	Identification of convergences and divergences across cases to develop a final interpretative account (14).	Themes were systematically compared across participants and refined through collaborative discussion to develop the final superordinate themes.

Although IPA has most commonly been applied to individual interview data, it has also been used with focus-group data. This application is methodologically debated because accounts generated in focus groups are shaped partly through interaction with other participants, potentially creating tensions with IPA’s idiographic emphasis on individual meaning making (21–23). However, IPA can be adapted to focus group data when the authors consider how the group setting influences individual accounts (22). In this context the hermeneutic process can take on an additional dimension. Rather than solely involving the “double hermeneutic,“in which authors make sense of participants making sense of their experiences (14), focus group interaction can involve a “multiple hermeneutic “in which the researcher makes sense of participants making sense of both their own experiences and those shared by others (22).

In the present study, focus groups were chosen because interaction between breast cancer survivors could prompt recollection, comparison, elaboration, and reflection on experiences of late effects and survivorship. The group was therefore understood as the context in which individual accounts were expressed and developed, rather than as the primary unit of analysis. The analysis retained an idiographic orientation by tracing and examining each participant’s contributions separately before moving to patterns across participants.

Both interviews were audio-recorded. The first three authors listened to the audio and read the transcripts multiple times to become familiar with the data. They independently examined each participant’s contributions across the focus-group transcript, coding statements with attention to how each woman described and made sense of her experiences. Preliminary themes were developed for each participant before the individual accounts were compared to identify similarities and differences across participants. Attention was also given to the interactional context in which accounts were produced, including instances in which participants prompted, affirmed, extended, or contrasted one another’s experiences.

The authors subsequently compared their coding and interpretations through collaborative discussion, returning to the transcripts when interpretations differed and refining the themes through discussion. The fourth author contributed to the interpretative discussions in a supervisory role. Only after the individual accounts had been examined and compared were themes considered across the two focus groups. In this way, the analysis sought to preserve IPA’s idiographic commitment while recognizing that participants’ accounts were generated within a socially interactive and partly co-constructed context.

The interviews were conducted and transcribed verbatim in Faroese, and the analysis was undertaken using the Faroese transcripts. Only quotations selected for presentation in the manuscript were subsequently translated into English by the first three authors. Translation prioritized the preservation of meaning and context rather than relying solely on literal translation. Where Faroese expressions did not have a direct English equivalent, alternative translations were discussed among the authors to reach a contextually appropriate English rendering.

All participants were pseudonymized, and identifying information was removed from the transcripts to ensure confidentiality and anonymity.

### 2.6 Methodological and ethical considerations

Consistent with the interpretative orientation of IPA, reflexivity was integrated throughout the research process. The authors recognized that data generation and interpretation were shaped by their professional backgrounds, prior knowledge, and interactions with participants. The first three authors are registered female nurses with backgrounds in surgical nursing, older-person care, management, and primary healthcare. The fourth author is a psychologist and university lecturer with experience in qualitative research and supervised the research project. These backgrounds informed the authors’ pre-understandings of breast cancer, treatment, care pathways, and late effects. Gadamerian concepts of pre-understanding and openness informed the authors’ reflexive stance: rather than attempting to set prior understanding aside, assumptions and interpretations were critically discussed throughout data collection and analysis while remaining open to participants’ accounts challenging and expanding existing understandings (15).

Reflexivity was supported through ongoing discussions within the research team concerning author roles, interview dynamics, pre-understandings, and possible influences on data generation and interpretation. Following the interviews and throughout the analysis, the authors discussed their assumptions, professional pre-understandings, role distribution, and alternative interpretations, returning to the transcripts when interpretations differed.

Attention was given to relational proximity, which is especially relevant in a small and interconnected society such as the Faroe Islands, where participants and authors may encounter one another across multiple social and professional contexts (12). In one case, a participant and one author were previously acquainted through the author’s personal life, although they did not have a close personal relationship. To reduce the potential influence of this prior familiarity, initial contact was handled by another author, and the acquainted author assumed an observer role during the interview. The prior familiarity was subsequently considered as part of the interpretative context.

The interconnected nature of Faroese society also presented ethical challenges related to confidentiality and deductive disclosure. Even when direct identifiers are removed, combinations of information concerning occupation, family circumstances, treatment history, or other contextual characteristics may enable recognition. To reduce this risk, participant characteristics were reported in aggregate, direct identifiers were removed, and potentially identifying contextual details were omitted or altered in quotations and descriptive material. Care was taken not to combine multiple individual characteristics in ways that could facilitate recognition. These decisions were made while seeking to preserve the meaning and contextual integrity of participants’ accounts.

As defined by the Faroese Act on Research Ethics Review of Health Research Projects Section 2, the study does not constitute a health research project but is considered to be an interview-based study.

The study can therefore be initiated without approval from The Committee on Health Research Ethics for the Faroe Islands (project reference F-26/24439).

The study was conducted in accordance with relevant ethical principles, including the Declaration of Helsinki (24) and the Code of Research Ethics of the University of the Faroe Islands. Participants received written and oral information about the study and provided written informed consent prior to participation. They were informed of their right to withdraw at any time without consequences and were encouraged to respect the confidentiality of information shared within the focus groups. Data were pseudonymized, identifying information was removed from the transcripts, and data were stored securely in accordance with the requirements of the Faroese Data Protection Authority (25).

## 3 Results

Four overarching themes were identified and are presented in the following section: 1. *Fatigue Sets the Agenda for Everyday Life*; 2. *Transitioning from Supported Patient to Abandoned Survivor*; 3. *A Disruption and Reconstruction of the Sense of Self*; and 4. *Invisible Late Effects and Social Misunderstanding*.

### 3.1 Fatigue sets the agenda for everyday life

Participants across both focus groups described a range of persistent late effects following breast cancer treatment which pertained to physical, emotional and cognitive functioning in their daily life. The physical late effects mentioned were, e.g., sensory disturbances in the hands and feet, radiation-related tissue damage, osteoporosis, loss of appetite, balance issues, sleep disturbance and joint pain in the axilla region, hips and knees. The cognitive late effects were forgetfulness, lack of concentration and sensitivity to outside stimuli such as noise, light and temperature. Concerning the emotional late effects, fear of cancer recurrence was mentioned, however, the participants primarily talked about feeling abandoned by the health care system after concluding treatment with inadequate information about follow-up care, which left them in a state of “*shock”, “lost”* and *“lonely”*, followed by a demanding process of reconstructing their sense of self and their daily lives. Fatigue, however, was repeatedly highlighted as a consistent late effect that set the agenda for everyday life, shaping participants’ daily routines, professional and social lives, as well as influencing decisions about how they allocated their time and energy.

During the interviews, participants described treatment-related tiredness as persistent and “*draining*, “ to the point of being “*indescribable*” to those who had not personally experienced this kind of exhaustion, as illustrated by the following accounts:

“Yes, and then it is this tiredness all the time … draining. For me it’s draining you know … for energy…” (Participant 7)

“[ … ]. And then there is fatigue, it is completely indescribable, you know. Yes.” (Participant 5)

*“It is very hard to describe how tired you are” (Participant 3).*

These statements were expressed with particular emphasis, suggesting that fatigue resulting from cancer treatment differs from ordinary tiredness. This fatigue extends to both physical and emotional functioning and is described as something that must be continuously *“stabilized”* to *“exist all the time”* as illustrated in this exchange by participants and the moderator:

“You just got to stabilize the psychological and physical energy and try to just exist all the time.” (Participant 6)

“Yes, sometimes I really feel like it is all about just existing” (Participant 7)

*Moderator: “that the energy is used for that? Existing?”*.

*Participants responded affirmatively.*

By emphasizing that their energy must be largely *“used for just existing, “* it becomes evident that fatigue is experienced as a late effect that consumes much of the participants available energy and limits their ability to engage in everyday life and complete daily tasks as they had prior to treatment. One participant explained how she used to be someone who “*could do anything”*, and how this image of herself had changed after cancer treatment:

“[ … ] you used to be a someone who actually could do anything, and had energy for everything and things like that and … [ … ]” (Participant 5)

This experience of being *less capable* post-treatment echoed throughout the interviews. Other participants further described how activities, such as household chores, which had previously been done with ease, were now experienced as physically demanding, difficult or at times impossible to complete due to fatigue:

“From being able to cook dinners and wash and clean … I can’t wind a washcloth now. Not at all. I need help to wash the house. I can vacuum, but when I’m done in one room, I’ve got to wait at least one or two whole days before I can take more [rooms]. I can’t do it. If I am to clean a whole house, then I’m exhausted for a whole month. I am of no use. Fatigue … yes, it is a kind of fatigue that … I don’t know … I’ve never run a marathon, but I can promise you, you know…” (Participant 3)

Comparing everyday household chores to *“running a marathon”* and being exhausted for days, or even a month later, illustrates how fatigue plays a fundamental role in shaping participants daily lives after treatment, influencing what they can do, when they can do it and how they plan on doing it.

Although fatigue was described as present *“all the time, “* participants explained how their energy levels could fluctuate throughout the day:

“[ … ] I am at my best in the afternoons [ … ]” (Participant 3)

“You say [addresses other participant] that you are no good in the morning, but that is when it is best for me. [ … ]” (Participant 5)

In response to this fluctuation in energy levels and anticipated exhaustion following daily tasks, participants learn to *“work around”* their fatigue:

“[ … ] I work around [the fatigue] somehow. [ … ] because odds are that some mornings I won’t have the energy, no chance that I have the energy before eleven o’clock. That is also completely fine. And I also know that, that on normal days you know, I am useless before 12 o’clock, I can manage but I am not very useful. If I decide to do something anyway [ … ] then I know to not make any plans for the next day. That is best for me. I can of course manage two days, but then I crash completely on the third day, then I am useless. [ … ]” (Participant 2)

“But if I don’t have the energy to pick up the vacuum cleaner today, well, then it’ll be tomorrow of the day after [ … ]” (Participant 7)

This suggests that participants learn to adapt to the persistent fatigue in their everyday lives. Fatigue thus seems to be experienced as something that requires planning and energy management, to avoid prolonged exhaustion following activity.

Fatigue also had an impact on participants’ professional lives. Many participants described leaving employment or entering early retirement, and those who remained in paid employment describe being *“completely exhausted”* when coming home and require recovery time afterwards, as explained by this participant:

“[ … ] And I am very happy with that, but I have no energy. I have no energy to work at all. But I have chosen to anyway. So the days when I am at work, I lie completely exhausted when I come home again. [ … ] (Participant 7)

Because fatigue often followed periods of activity, participants described being cautious when committing to future plans, which by this participant is experienced as *“very frustrating”* due to a sense of letting others down:

“I don’t dare to volunteer. I don’t know if I am fine on Tuesday this week or Thursday next week. That’s it. It is very frustrating to say ‘you know what. I haven’t got the energy after all. I’m sorry, but I can’t.’ (Participant 3)

By *“not daring”* to commit due to uncertainty about their future energy levels, fatigue appears to not only have a personal influence on participants’ lives but also extends into influencing their social lives.

As participants describe how they organize their lives around their exhaustion, it becomes evident that they adopt different strategies for adapting to fatigue. One participant illustrated that pushing beyond her limits could result in a *“crash, “* leading to prolonged exhaustion lasting several days:

“[ … ] if you push it for too long, then … you crash, then it takes several days until you get back up again you know … it’s this limit that … there is no use in fighting it. No you just crash” (Participant 5)

By *“not fighting it, “* she appears to have learned to accept fatigue as an ongoing late effect in her life. Another participant, on the other hand, described how she continues to *“fight it, “* illustrating a different way of relating to the persistent fatigue.

“ [ … ] but I try and fight it.” (Participant 7)

Rather than representing opposing positions, these accounts reflect different ways of adapting to their fatigue. Whether through resistance or acceptance, fatigue is experienced as a late effect that must be continuously negotiated and managed, illustrating how participants develop resilience while learning to live within the limitations of their changed bodies.

These accounts illustrate how fatigue, caused by cancer treatment, sets the agenda for survivors’ everyday lives following illness. Fatigue, rather than being experienced as a single late effect among many, appears to have a significant influence on how survivors structure their daily lives and allocate their time and energy, both on a physical, emotional and social level. Fatigue further extends into influencing survivors’ social and professional lives, determining whether (and how) they will engage in daily activities or employment. Thus, fatigue can be seen as an all-encompassing late effect which continuously sets the agenda for daily life after treatment, where breast cancer survivors learn and adapt their new bodily limitations, activities and commitments, as active management of the persistent exhaustion.

### 3.2 Transitioning from supported patient to abandoned survivor

While fatigue structures participants’ everyday lives post-illness, the conclusion of treatment introduces a different disruption. Across both focus groups, participants described how concluding cancer treatment created an emotional rupture, which acted as a catalyst for the demanding and ongoing reconstruction of everyday life following illness. The transition from being a cancer patient to a cancer survivor is marked by a sudden loss of professional support and heightened uncertainty about the future. This rupture can be interpreted as emotional late effects of treatment, which becomes embedded in participants’ everyday lives, highlighting the immediate responsibility of navigating life beyond treatment independently^1^.

The following quote, was expressed at the end of a broader life narrative describing changes in family, professional, and social life following treatment, illustrates how the conclusion of treatment was experienced as a demanding transition:

“So, it was very, you know, a constant balancing act, to find back on the track, so yes. Psychological diffi … I don’t know if you can call them difficulties, it was…. Aftereffects or long-effects, psychological long-term effects of all factors in life’s … all of life’s circumstances had changed, you know. Ehm. And I have nowhere to go. [ … ]” (Participant 6)

The expectation to go *“back on track”* post-treatment appears to be mismatched with the reality of *“all of life’s circumstances had changed”*, creating a sense of instability and discontinuity. The experience of having *“nowhere to go”* reflects a perceived sense of abandonment by the healthcare system, alongside a sudden shift towards sole responsibility for managing the aftermath of survivorship. This is further illustrated in the following quotes, where participants described feeling *“lost”*, *“weird in the head”* and in *“shock”* following treatment, which appeared to be linked to a sudden withdrawal of structured support and a lack of information regarding follow-up care:

“[inhales] I don’t know, maybe to get an explanation on certain things. You are quite lost in many ways, and also what are your rights and so on. You get no information about that, nothing at all!” (Participant 3)

“I think you are in such a state of shock when [treatment ends] … [ … ] you feel so weird in the head, you are unable to seek out anything [support] at all” (Participant 2)

These descriptions illustrate how concluding treatment alongside the absence of structured support creates emotional ruptures which disrupts participants’ sense of continuity between their pre-treatment lives and their anticipated futures. This disruption may also be understood as a shift in identity, as participants transition from being *“patients”* within a structured system to being positioned as *“cured, “* and therefore expected to independently manage life after cancer treatment, often without adequate information or support in how to do so. Concluding treatment thus appears to represent an abrupt turning point in participants’ lives, where life is experienced as divided into two distinct phases: a life before and a life after breast cancer, as illustrated by this participant:

“[ … ] so, there is actually a life before and definitely a life afterwards. [ … ]” (Participant 5)

One participant illustrated this transition metaphorically, describing herself as a *“cotton ball”* moving through a *“cotton tube”* during treatment:

“I felt exactly like I was this cotton ball being sent through a cotton tube—it was so soft and gentle. Every time you hit a wall, it was just a soft landing, and you were gently sent on again. And then when the treatment came to an end, you reached the end of that tube, [ … ] (long pause, she is emotional) … And then you’re standing on the edge right there, and you look in, and everything is just empty and black. You must take the first step out there, and you have no idea where you’re turning or whether there is anything underneath, or beside you, or anywhere. [ … ]” (Participant 6)

The *“cotton tube”* becomes a metaphor for the healthcare system, representing safety, predictability, and support during active illness. When treatment ended, this protective structure was abruptly removed, leaving the women feeling alone, abandoned, and exposed in the aftermath of a traumatic experience, further highlighting the emotional late effects associated with surviving cancer. She further compared the completion of treatment to the receding of a tsunami, where everything is left in ruins and survivors are left to rebuild their lives on their own:

“The first phase is when you receive the diagnosis, and everything is chaos, and that is when the tsunami comes crashing in, where everything in its path is destroyed, and everything lies… (gestures with arms). And the next phase is when the tsunami recedes and that is the treatment when you … You experience a relief, you know. However, the third phase is what remains after the tsunami has … yes everything is destroyed and you must clean up. And it is in this phase where we are dropped in this black hole, and we are actually standing alone and must … figure out how to piece everything back together again, you know?” (Participant 6)

The aftermath of a tsunami emerges as a metaphor for after the end of treatment, illustrating how survivorship was not experienced as a return to normality, but rather as entering an uncertain and unfamiliar future, in which the women are left to independently navigate the aftermath of surviving cancer and reconstruct their everyday lives following treatment.

The period following treatment was described as emotionally more distressing than receiving the diagnosis itself. One participant described this transition as the *“worst part”* of the entire illness trajectory, where the phrase *“all the afterwards”* can be interpreted as encompassing the unstructured and uncertain period following treatment, marked by a lack of support:

“But it was more this part here—when the treatment is finished, what then!? Then you’re told at the oncology department that you should just let them know if something happens, and yeah … that feeling there … From becoming ill and basically, they are over you all the time … if you feel a small twinge, it gets checked … and then suddenly you are just at home alone with nothing to go back to. (long pause) I actually think that was the worst part. It was terrible to be told that you were ill, but… (long pause) … all the afterwards.” (Participant 7)

She further described how completing treatment was accompanied by a strong sense of panic and loneliness:

“[ … ] (inhales)… I think that was when I really panicked, when the chemotherapy ended … I simply felt so alone… (long pause) [ … ]” (Participant 7)

Another participant recognized and affirmed this experience, further describing it as the *“darkest feeling”* and expressing how she also felt *“terribly alone”*:

“Oh yes, that hit me right in a certain place, I felt exactly the same! (long pause) [ … ] And then you felt after the treatment, you’re standing there and you have to build and blindly find, where the walls are. If there is any light? Can you hold onto something? And you just feel, as you said [gestures other participant] it is the darkest feeling that … terribly lonely” (Participant 6)

Both participants became visibly emotional during this exchange, reflecting a shared emotional experience. Another participant described how she experienced an inability to *“allow herself to react”* during treatment, highlighting a shift in her emotional processing following its completion:

“[ … ] I could not allow myself to react. I had to try and stay strong for everyone else all the time. So it is extremely changed psychologically. I don’t think it is until you have finished that … now I was finished, and that was very … you have been in it for so long and all that … [ … ].” (Participant 5)

This suggests that some emotional responses may be deferred during treatment, as participants must *“stay strong for everyone else”*, and begin to surface following the conclusion of treatment. Thus, the end of treatment marks not only a clinical transition, but also an emotional release, highlighting how the emotional impact of cancer may unfold more fully after completion of treatment rather than during the illness itself.

Concluding treatment and being discharged from the health care system, therefore appears to mark a significant turning point in participants’ lives, representing not only the end of treatment but the beginning of a new and uncertain phase. This moment can be understood as the starting point of their “new life, “ where participants feel they are independently required to navigate everyday life without the structure and security of ongoing care. In this sense, discharge does not signal a return to normality, but rather the first step in learning how to live differently as a cancer survivor. Consequently, survivorship appears almost anticlimactic, as the conclusion of treatment does not bring the anticipated sense of closure or relief, but instead reveals the extent of the emotional late effects along with the complexity involved in surviving cancer and the ongoing process of reconstructing everyday life thereafter.

### 3.3 A disruption and reconstruction of the sense of self

As participants transition from supported patients to abandoned survivors, this emotional rupture extends into shaping their sense of self. Across both focus groups, participants described being fundamentally changed post-treatment followed by an ongoing and often demanding process of learning to navigate life after treatment, as physical, emotional and cognitive late effects continue to shape everyday life. Although participants expressed gratitude for being cancer-free, this was accompanied by sudden and often unexpected feelings of being *“so very much changed*” after treatment, as illustrated by the following accounts:

“Yes well … you are very affected afterwards. [ … ] I think you are just so very much changed that…” (Participant 5)

“Yes very, very. Everything is rearranged and everything is changed. Everything is completely rearranged you know” (Participant 7)

This change reflects a disruption in participants’ sense of self, manifested through physical, emotional and cognitive functioning as illustrated in the following accounts:

“[ … ] and then it’s these late effects, it is my feet that is the worst. Because of it I have difficulties wearing shoes. [ … ] and a little in my hands. I must be careful how I grab things, because sometimes it just falls out of my hands.” (Participant 1)

“[ … ] When my head finally says ‘ah you are holding something’ then I’ve already lost it, you know? It is frustrating, extremely frustrating. [ … ].” (Participant 3)

“[ … ] and I had changed cognitively. I felt that really well.” (Participant 6)

A contributor to this change and disruption of the self appears to be a loss of trust in bodily and cognitive abilities, resulting in feelings of frustration, as these changes require ongoing active management. This disruption also affects participants’ professional and familial roles, as well as their relationship to previously meaningful hobbies as illustrated by these quotes:

“[ … ] I’m on early retirement. I mean, I couldn’t just be on sick leave; I couldn’t go back to work at all. It’s just that you are so damaged by chemo and radiation. [ … ]” (Participant 5)

“I only work one day a week now … that’s okay.” (Participant 4)

“Yes, I work part time after treatment, so now I only work one day a week.” (Participant 2)

“[ … ] And it’s terrible to lie there and look at all the yarn and needles, because I … I don’t have the energy. [ … ] It’s really hard, yes, that you can’t use your hobbies like that … because that’s what I’m good at, using my hands.” (Participant 3)

No longer being able to return to previous professional roles or engage in meaningful activities appears to create a sense of loss in their identities, suggesting a process of grieving the former self, which further highlights the emotional late effects of cancer treatment.

However, it is further explained how this disruption initiated a process of *“needing to get to know herself again”* after *“becoming someone else”* post treatment - something this participant had neither anticipated nor been prepared for:

“But it bothered me that I had become someone else. But I didn’t know. I didn’t know who I was, because I had only reached this point [in my life]. I just needed to learn to know myself, I didn’t even know that I had to get to know myself [after treatment].” (Participant 6)

She continues:

“So, it was very much about also, finding out ‘who am I now?” (Participant 6)

*“Finding out”* who they are suggests that the reconstruction of a new self begins in the aftermath of this disruption. This is not necessarily because the late effects disappear, but rather because participants learn to adjust to their altered bodies and capabilities as these accounts illustrate:

“What I think is the worst is that I can’t walk so well. [ … ] I have no idea if the foot is standing on the floor or where it is you know? So, I spend many days … looking down to see if the foot is down, listen if I can hear the foot is down [laughs] [ … ] probably just the head that needs to learn anew.” (Participant 3)

“I get this discomfort in my feet, got it quite quickly [after treatment] and I have learned to live with it. It is very hard, but I have learned to live with it.” (Participant 1)

As time progresses, participants described a gradual movement towards accepting their new realities following treatment, although this process often required letting go of previous expectations of themselves and accepting new limitations. One participant described it as an internal struggle which involved a reevaluation of *“who she is”* and of her new capabilities.

“And it was a terrible struggle inside. “Should I quit my job permanently and just continue on this track with finding out who I am, and what I am capable of and what I have the energy for and how we can find a balance in all this?” (Participant 6)

There seems to be a gradual transition from resistance toward adaptation where reconstructing the self begins to take shape, involving learning to listen to the body and recognizing new limits.

“You need to pay attention to your body [ … ] it is a new way to live. Because that’s how it is. It is no use to fight it. No.” (Participant 5)

“I think you also learn to listen to your body.” (Participant 1)

This process of reconstruction appears as a forced and necessary adaptation rather than an intentional transformation. This adaptation can be understood as a demanding transition involving continuous planning and prioritization.

“It is about learning what … because it demands a lot of me [ … ] So it is always about prioritizing and planning.” (Participant 5)

“[ … ] I mean, I don’t care if I sleep in the living room and people come in, I don’t care. Because that is my way of doing it, to fall asleep. [ … ] that is my therapy, to recover” (Participant 3)

These accounts illustrate how reconstruction involves developing new strategies for managing social expectations and prioritizing self-care in everyday life. During the first focus group interview, the moderator asked whether knowing one’s boundaries was important:

Moderator replies: “so it is about knowing your boundaries?”

All participants (in unison): “mhm yes”

This collective affirmation highlights the importance of boundary-setting, suggesting that reconstruction of the self involves an ongoing negotiation between personal limits and external expectations. Not only because of new physical and cognitive changes, but because time itself has been given a new meaning as illustrated by this participant:

“[ … ] because time has also been given a new perspective [ … ]”(Participant 6)

Faced with their own mortality, participants describe a shift in how they value and allocate their time, prompting a re-evaluation of what is meaningful and worthy of investment. Some participants even expressed a sense of personal growth emerging from adversity as this participant explains it:

“I don’t know, I want to say, that despite all this hardship you go through, I’ve become a better person [laughs lightly] [ … ]” (Participant 2)

This suggests that reconstruction is not solely about adapting to new limitations, but also about redefining what constitutes a meaningful life after cancer, thereby revealing how late effects extend beyond bodily symptoms to influence participants’ orientation toward time, meaning, and life priorities. Reconstruction thus appears not as a return to a previous self, but as the development of a new and sustainable sense of self within altered physical and emotional boundaries.

In conclusion, the reconstruction of the self following cancer treatment appears as neither linear nor complete, but as an ongoing process shaped by the enduring presence of physical, emotional and cognitive late effects affecting their new identities on a personal and professional level. Participants do not simply *“return”* to who they were before illness but go through a continuous process of renegotiating their priorities and new realities, where new boundaries must be recognized and respected in their everyday lives. Reconstruction thus reflects both vulnerability and resilience, in the process of creating a new and meaningful way of being after surviving cancer.

### 3.4 Invisible late effects and social misunderstanding

This disruption and ongoing reconstruction of the self is often invisible to participants’ social surroundings. As they attempt to adapt to their changed bodies and identities, they encounter a social world that often fails to recognize these internal changes. Across both focus groups, participants described a mismatch between how they *felt internally* and how they were *perceived externally* by others:

“[ … ] ‘You look so good.’ On the outside, but inside everything is a mess … I usually say ‘you don’t see me on the inside.’” (Participant 7)

Here, the phrase *“you look good”* becomes a form of unintentional invalidation. While others interpret her appearance as a sign of recovery, she experiences an ongoing internal *“mess”*. Another participant further described how surviving cancer and living with the late effects of treatment appears largely invisible to others and therefore places her *“up against”* the expectations of the outside world:

“… you look at us, but no one sees that we’ve had cancer, or that we’ve received treatment or are currently receiving treatment. And that is also what you are up against all the time. You know the world is watching you and they see “but you are healed now. You do not look sick. You have received treatment. You look good. What is wrong with you?”“ (Participant 6)

The contrast between *“outside”* and *“inside”* highlights an incongruence, between lived reality and the image presented to the world, where being seen as “healed” creates pressure to function normally and thereby generating misunderstandings within their social surroundings.

There was a common agreement during both interviews, that it is hard to explain what life is like after cancer treatment to others who have not personally experienced it, as illustrated by these accounts:

“It’s so strange … it’s so indescribable … you must experience it. It’s hard to explain it to those who haven’t had [cancer treatment]. But all of you sitting here, you understand what I’m talking about.” (Participant 5)

“…no one understands it, it is such a terrible loneliness … You try to explain … no one understands it. Well yes, you understand part of it (turns to another participant) and I understand you, because we both have … but there is no forum where you can really…” (Participant 7)

The social misunderstanding of life after cancer treatment and its late effects can be experienced as *“indescribable”* to others, which can create a sense of *“terrible loneliness.”* The absence of a safe space, such as a forum, where one can feel understood and validated, is further contributing to these feelings of loneliness.

One participant explains how she becomes *“drained”* in social gatherings and by changes in her immediate physical environment:

“Yes completely. You become completely drained. Also sensitive to heat and cold … very sensitive to changes in the environment. I imagine it must be like when you are born prematurely. All the senses are immature, and the chemo has opened everything up and you must … I don’t know…” (Participant 6)

Comparing her felt condition to being born prematurely with all her senses being *“immature”* suggests that participants experience their changed bodies as vulnerable and hypersensitive to their surroundings following treatment, further highlighting how consequences from cancer treatment are largely invisible to others and becomes an internalized bodily experience.

Participants described how treatment-related fatigue and the need for rest was often misunderstood by others, requiring them to defend their limitations. One participant recalled being judged for resting during the day:

“…so I’m lying on the sofa in the middle of the day, and my son-in-law comes in and says ‘damn, you’re just lying here on the sofa’… That’s just how it is – no one understands it, you know? He comes with a ‘get up and make yourself useful’.” (Participant 7)

The necessity for rest is here interpreted as laziness. Fatigue thus becomes something that must not only be managed physically, but also socially justified. Same participant explains how she withdraws socially due to the physical, emotional and cognitive strain it can cause and only attends social events in which it would be socially unacceptable to not attend, even though she *“dreads”* it because she knows it does something to her that *“isn’t so good”*:

“I think you withdraw socially as well. I’m not going to any parties or anything. I must attend something in a few days, but I dread it really … but I’m being voluntarily forced, I need to attend [a large social event] … yes.” (Participant 7)

She continues:

“[ … ] I don’t like to … be in a group of people anymore. Because I know that it does something to me that isn’t so good. Yes. And then I just withdraw and don’t talk to anyone. I just sit and study the noise and people talking between themselves and such. Yes. That is actually some of the worst I think.” (Participant 7)

This statement illustrates how participants go through a continuous negotiation between their bodily and emotional limitations and the perceived social obligation to participate in everyday social life. Another participant further explains how she feels she is letting others down, if she has committed to help with a social event, but must cancel due to being exhausted that day.

“You feel like such a let down, you’ve promised but you can’t deliver. That is terribly hard.” (Participant 3)

Feeling like a *“let down”* illustrates an internal struggle where bodily limitations interfere with the desire to maintain social relationships and fulfil social expectations.

These accounts illustrate how late effects following cancer treatment can be invisible and can create misunderstandings in participants’ everyday social contexts. Although survivors may appear physically recovered to others, they continue to experience ongoing internal challenges that remain largely unseen. This mismatch between external appearance and internal experience can lead to internal and external pressure to function normally as prior to cancer, and the need to repeatedly justify one’s limitations. Survivors describe having to navigate a complex social landscape in which they must continuously negotiate between their bodily limitations and the expectations of the outside world.

## 4 Discussion

This study explored which late effects are experienced by female Faroese breast cancer survivors following treatment, and how these impact everyday life. Using IPA to analyze data from two semi-structured focus group interviews with a total of seven participants, four themes were identified: (1) Fatigue Sets the Agenda for Everyday Life, (2) Transitioning from Supported Patient to Abandoned Survivor, (3) A Disruption and Reconstruction of the Sense of Self, and (4) Invisible Late Effects and Social Misunderstanding. These findings provide insight into how late effects become embedded in survivors’ everyday lives, persisting for weeks, months, and years following treatment, and shape their physical, emotional, and social functioning.

The findings demonstrate that survivorship is not experienced as a return to normality, but as an ongoing and demanding process characterized by fatigue, emotional disruption, changes in identity, lack of systematic support, and pressure from external expectations to function as prior to treatment. The findings are consistent with existing research where survivors report a wide range of persistent late effects following treatment, including insomnia, neuropathy, shoulder impairment, cognitive impairment, fear of cancer recurrence, balance issues, chronic pain and musculoskeletal symptoms, all of which affect their daily lives, with fatigue appearing to be among the most burdensome (26–29). Participants’ descriptions in this study of increased sensitivity to external stimuli, are consistent with a previous study where respondents reported hypersensitivity to cognitive stimuli which caused burdensome symptoms (30). Experiences such as loss of appetite may reflect gastrointestinal symptoms (28). The qualitative studies highlight that survivors experience ongoing bodily and emotional challenges that require continuous and active adaptation in everyday life (30, 31).

### 4.1 Fatigue sets the agenda for everyday life

Fatigue was identified as an all-encompassing late effect that shaped participants’ everyday lives, influencing their ability to engage in household chores, employment, and social activities. This finding is supported by existing literature, where survivors report reduced functioning and work capacity due to fatigue (28, 30, 32). In this study, fatigue was described as persistent, unpredictable, and requiring continuous energy management.

### 4.2 Transitioning from supported patient to abandoned survivor

Participants described the end of treatment as an abrupt and emotionally challenging transition, characterized by a loss of structured support, uncertainty, and feelings of abandonment by the healthcare system. This finding is consistent with qualitative research in which survivors report feeling unprepared for life after treatment and experiencing a lack of follow-up care, where access to relevant information, guidance, and ongoing support is identified as essential for managing everyday life challenges (30, 31). Participants in this study also expressed expectations of getting “back on track” following treatment, similar to their lives prior to diagnosis. This is consistent with existing literature, where survivors commonly expected to live their daily lives as before their diagnosis (30).

### 4.3 A disruption and reconstruction of the sense of self

Participants described survivorship as a process characterized by identity disruption followed by a reconstruction of the sense of self. This appears as an ongoing process shaped by the enduring presence of physical, emotional, and cognitive late effects, which influence participants’ identities on personal and professional levels. These findings are consistent with existing literature, where breast cancer survivorship has been associated with reduced work capacity (32). The findings further suggest that participants engage in a continuous process of renegotiating priorities and adapting to new realities. Reconstruction of the self thus reflects both vulnerability and resilience, as participants strive to create a new and meaningful way of being after cancer treatment. This is supported by previous research, which shows that survivors actively work to maintain a meaningful everyday life despite reduced mental and physical capacities (30).

### 4.4 Invisible late effects and social misunderstanding

The findings suggest that late effects following treatment are often invisible to others, creating a mismatch between survivors’ internal experiences and how they are perceived externally. This may lead to pressure to function as before treatment, as well as a need to justify one’s limitations. Participants described withdrawing from social interactions, which aligns with qualitative literature showing that survivors may no longer find the same value in social gatherings due to reduced physical and mental capacity (30). Furthermore, participants expressed a sense of loneliness, as late effects were described as difficult to articulate and often misunderstood by others. Previous research highlights the importance of open and supportive communication with close social networks in helping survivors manage ongoing challenges (30).

### 4.5 Survivorship in Faroese context

The challenges identified in this study are not necessarily unique to Faroese breast cancer survivors, as similar experiences have been reported internationally. What may distinguish the Faroese context is the small and socially interconnected nature of Faroese society, combined with the geographical and organizational characteristics of cancer care. Together, these contextual features may shape how the challenges of survivorship are experienced and navigated.

Close and overlapping social networks can contribute to a high degree of social visibility within small-scale societies such as the Faroe Islands (33). Participants described withdrawing from social gatherings as a consequence of their late effects. In this context, although the late effects themselves may be invisible to others, withdrawing from social activities or not attending expected gatherings may be highly visible and create a perceived need to explain or justify one’s absence. Within the socially interconnected Faroese context, where changes in social participation may be particularly noticeable, this may add another dimension to the social consequences of invisible late effects.

The organization of cancer care in the Faroe Islands has also developed during the period represented by participants’ experiences. The appointment of a resident oncologist in 2019 represented an important organizational change in local oncology provision (7), while the Faroese National Cancer Strategy introduced in 2022 places greater emphasis on rehabilitation, quality of life, psychosocial well-being, and multidisciplinary follow-up (34). As participants completed treatment across different periods, their experiences reflect different organizational contexts, and it cannot be assumed that survivors completing treatment more recently would experience the transition to survivorship in the same way.

Treatment often involves both the Faroese and Danish healthcare systems, requiring travel and temporary relocation during parts of the treatment trajectory (8), followed by a return to the Faroese healthcare system, which has fewer specialist resources. Participants generally perceived the medical treatment as satisfactory; however, they described long-term follow-up and rehabilitation as less structured. In this context, survivorship may be characterized by fragmentation, uncertainty, and unmet supportive care needs, as the loss of support associated with active treatment may also involve losing established connections with healthcare professionals and services abroad. Against this background, participants’ descriptions of moving from supported patient to abandoned survivor may be understood within a care trajectory that extends across geographical and organizational boundaries.

These findings nevertheless point to the importance of developing survivorship care that is responsive to the geographical and organizational conditions of the Faroe Islands. Rehabilitation initiatives such as Iceland’s Cancer Rehabilitation Center Ljósið (35) may provide a relevant model because of similarities between the two small island settings. In the Faroese context, structured survivorship pathways could include individualized information about treatment-related effects and available rehabilitation, as well as greater continuity of information and follow-up across Faroese and Danish healthcare services.

Given the small population and healthcare system of the Faroe Islands, it may not be feasible to provide all highly specialized cancer services locally, and travel abroad for parts of cancer treatment may therefore remain necessary. Acknowledging this structural reality is important when considering how survivorship care can be organized within the Faroese context. The challenge may therefore lie not in eliminating cross-border care, but in ensuring continuity across geographical and organizational boundaries and supporting survivors as they transition from specialized treatment abroad to follow-up and everyday life in the Faroe Islands.

### 4.6 Strengths and limitations

A key strength of this study is its exploration of breast cancer survivorship in an under-researched small-scale societal context. The focus-group format enabled participants to compare, elaborate on, and reflect upon experiences shared by others, while the IPA-informed analysis maintained attention to individual meaning-making. Conducting both the interviews and the analysis in Faroese further enabled participants to express their experiences in their first language and helped preserve cultural and contextual nuances during analysis.

The study also has several limitations. The sample was small and self-selected. Recruitment through existing social networks, followed by snowball sampling, may have increased the likelihood of including survivors who remained affected by late effects or who were already engaged in support-seeking or survivor networks. Conversely, survivors who were less connected to support networks or other eligible participants may therefore have been underrepresented. These factors should be considered when assessing the transferability of the findings to the wider population of Faroese breast cancer survivors.

Participants had completed primary treatment at different time points, ranging from 2011 to 2024, and therefore represented different stages of the post-treatment trajectory (see **Table 1**). This variation is relevant to the interpretation of the findings, as both the experience of late effects and participants’ retrospective accounts may change over time. Time since treatment was not examined as a separate analytical dimension, and the small sample did not allow for meaningful comparison of patterns according to stage of survivorship. Consequently, the study cannot determine whether the nature or perceived impact of treatment-related effects differed according to time since treatment.

The two focus group interviews generated different interactional dynamics. The five-participant focus group provided a broader range of accounts and opportunities for participants to compare and respond to each other’s experiences. Although the second interview was dyadic, it provided a smaller and more intimate setting in which each participant had greater opportunity to elaborate on her individual account. Both participants engaged actively in discussing and reflecting on their own and each other’s experiences. Thus, while the dyadic interview offered less breadth of group interaction than the five-participant focus group, it provided greater opportunity for in-depth exploration of the two participants’ experiences. The interviews were therefore not considered interactionally equivalent but generated complementary accounts relevant to the research question.

The use of IPA with focus group data also presents a methodological limitation. IPA is idiographic in orientation, whereas accounts generated in focus groups are partly shaped through interaction and co-construction (22, 23). Although the analysis sought to preserve an individual focus by examining each participant’s contributions before identifying patterns across participants, it is not possible to separate individual meaning-making entirely from the group context in which the accounts were produced.

All participants had received endocrine therapy and described adverse effects associated with this treatment. However, information on whether participants were still receiving endocrine therapy at the time of interviews was not systematically collected. It was therefore not possible to determine the extent to which the symptoms described represented persistent effects of completed primary treatment, ongoing effects of endocrine therapy, persistent effects following previous endocrine therapy, or a combination of these. The findings should consequently be understood as reflecting participants’ experiences of treatment-related effects during survivorship rather than late effects attributable exclusively to completed primary treatment.

Although the analysis was conducted using the original Faroese transcripts, translating selected quotations into English may have resulted in some loss of linguistic or cultural nuance. For example, the Faroese term ‘*møði’* can convey tiredness, fatigue, exhaustion, or weariness depending on context; following discussion of its meaning within participants’ accounts, “fatigue” was considered the closest English rendering for this study. No formal back-translation procedure was undertaken.

Finally, the interconnected nature of the Faroese population also creates a risk of deductive disclosure, whereby participants may be identifiable through combinations of contextual information despite the removal of direct identifiers. Complete anonymity cannot be guaranteed in a small population, and the need to protect participants’ identities limited the level of individual contextual detail that could be reported. The findings are contextually situated within the Faroese healthcare and social setting and are not intended to be statistically generalizable, although they may offer transferable insights relevant to other small-scale or geographically isolated settings.

## 5 Conclusion

This study explored how female Faroese breast cancer survivors experience late effects following treatment and how these shape their everyday lives. The findings indicate that survivorship is not necessarily a return to normality, but an ongoing and complex process marked by persistent physical, emotional, and social challenges. The transition from supported patient to independent survivor was experienced as abrupt and emotionally challenging, highlighting perceived gaps in follow-up care.

Within the Faroese context, survivorship is shaped not only by individual experiences of late effects, but also by social, geographical, and organizational conditions, including care pathways that extend across the Faroese and Danish healthcare systems. These findings highlight the importance of structured survivorship pathways that provide individualized information, access to rehabilitation, and continuity of follow-up across geographical and organizational boundaries. Such support may facilitate a more gradual transition from the healthcare system and better prepare survivors to navigate everyday life after breast cancer.

## Data Availability

The datasets presented in this article are not readily available because the dataset consists of confidential interview transcripts. Due to participant anonymity and ethical agreements, the full data set cannot be shared publicly. Reasonable and limited requests for de-identified excerpts may be considered. Requests to access the datasets should be directed to BG-D, 2017.207@student.setur.fo.

## Footnotes

1 The Faroese term *“sálarligt”* encompasses both emotional and psychological aspects. In translating participants’ accounts into English, this term may be rendered as either *“emotional”* or *“psychological,”* depending on context. This theme will use the term *“emotional,”* when interpreting participants lived experiences while acknowledging its broader meaning, to avoid repetition.
